# Supplementary figures and images for: Exploring therapeutic strategies for infantile neuronal axonal dystrophy (INAD/PARK14)
Source: eLife. 2023 Jan 16;12:e82555. doi: 10.7554/eLife.82555 (PMC9889087; doi:10.7554/eLife.82555)

Figure 1- Source data 1

Figure 1A:

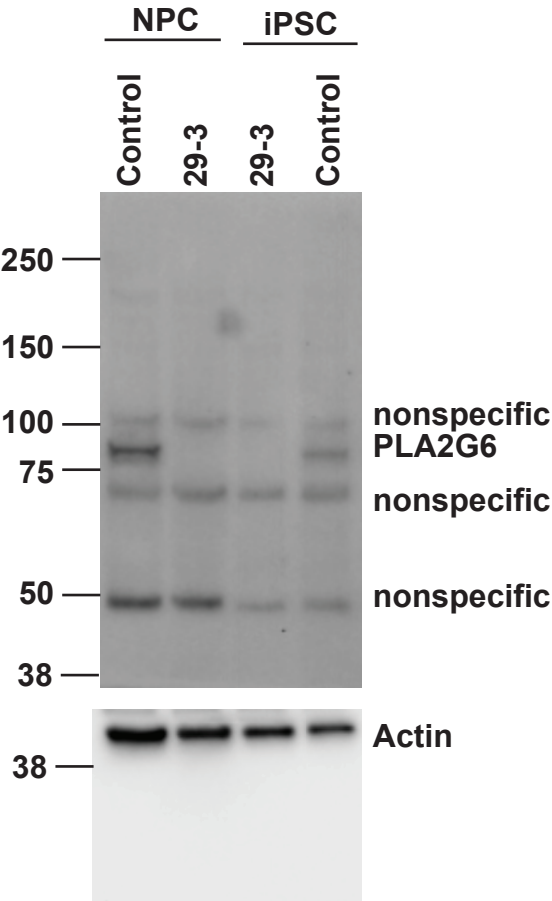

Supplement: Figure 1—source data 1. [file elife-82555-fig1-data1.pdf]

Figure 1- Source data 2

Figure 1F:

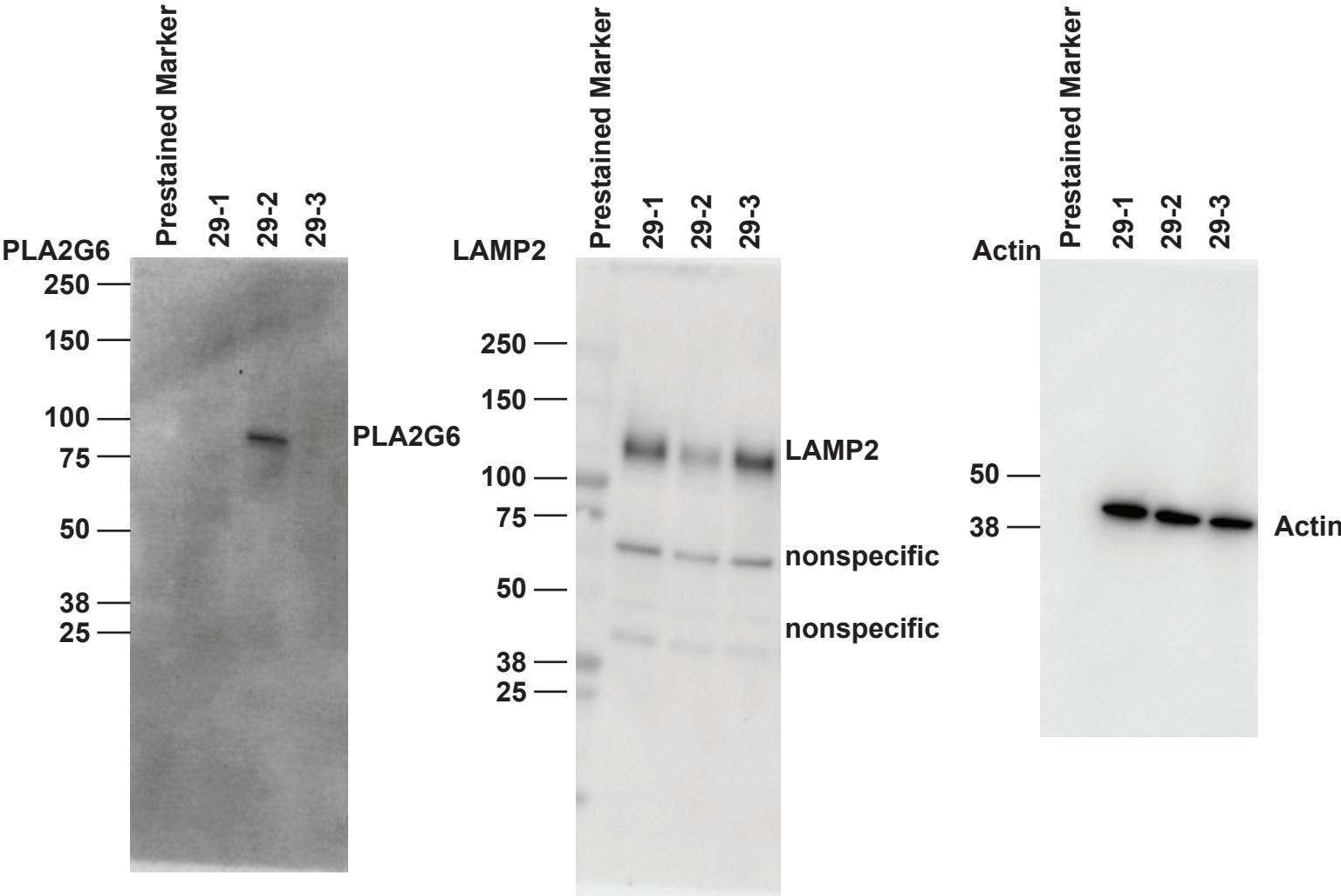

Supplement: Figure 1—source data 2. [file elife-82555-fig1-data2.pdf]

Figure 3- Source data

Figure 3C:

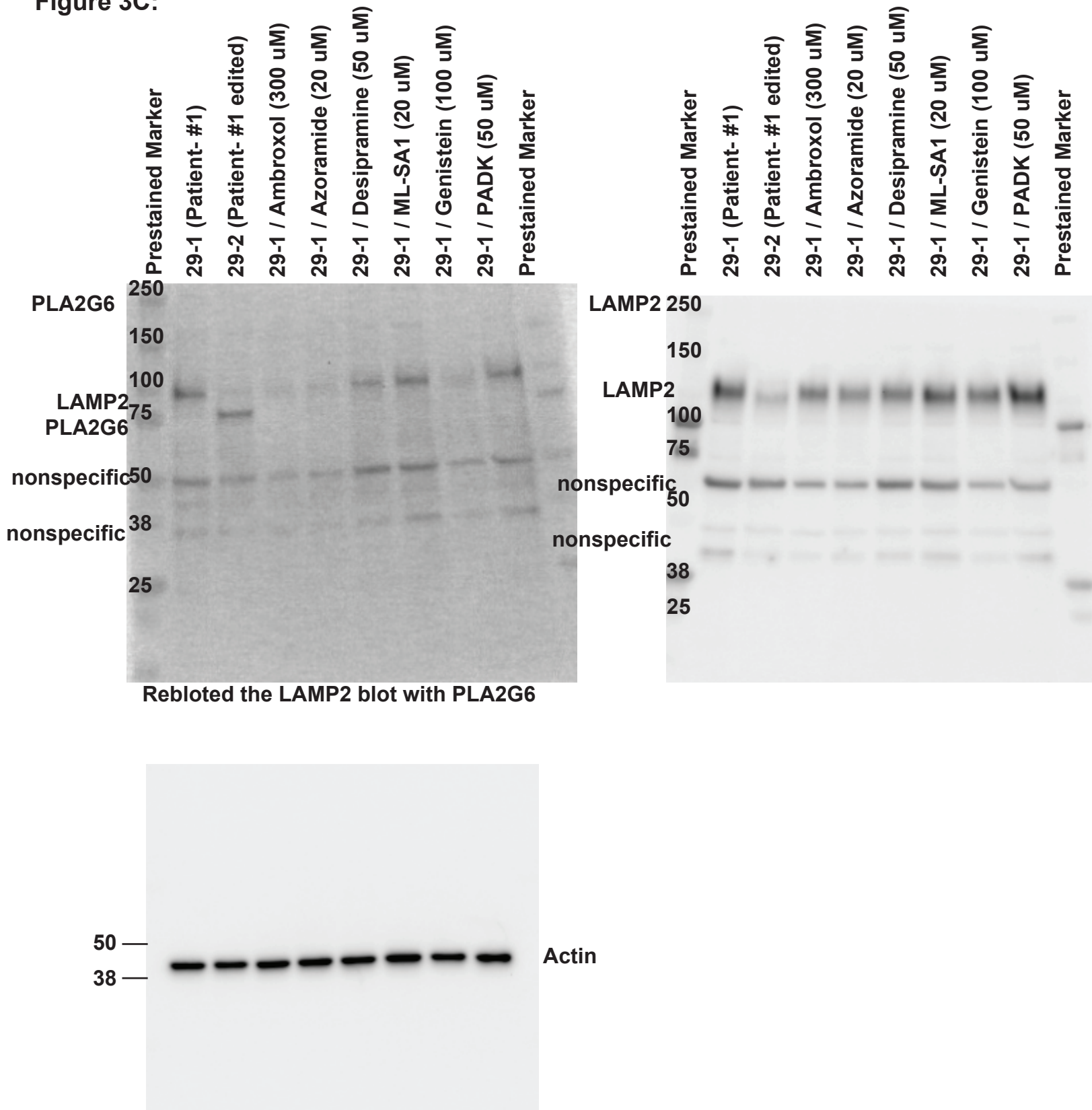

Supplement: Figure 3—source data 1. [file elife-82555-fig3-data1.pdf]

Suppl Figure 4- Source data 1

Figure 4B:

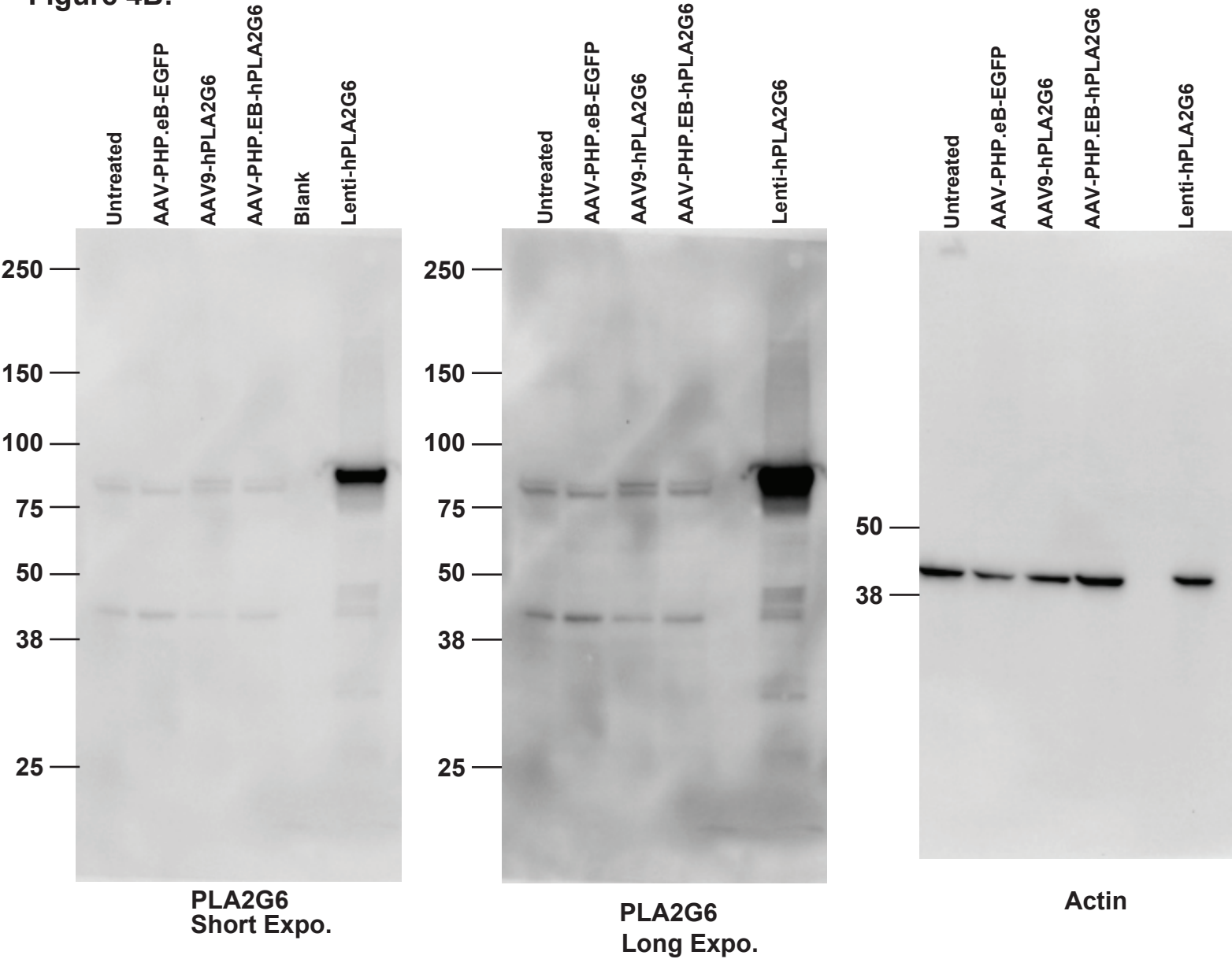

Supplement: Figure 4—source data 1. [file elife-82555-fig4-data1.pdf]

Figure 4C:

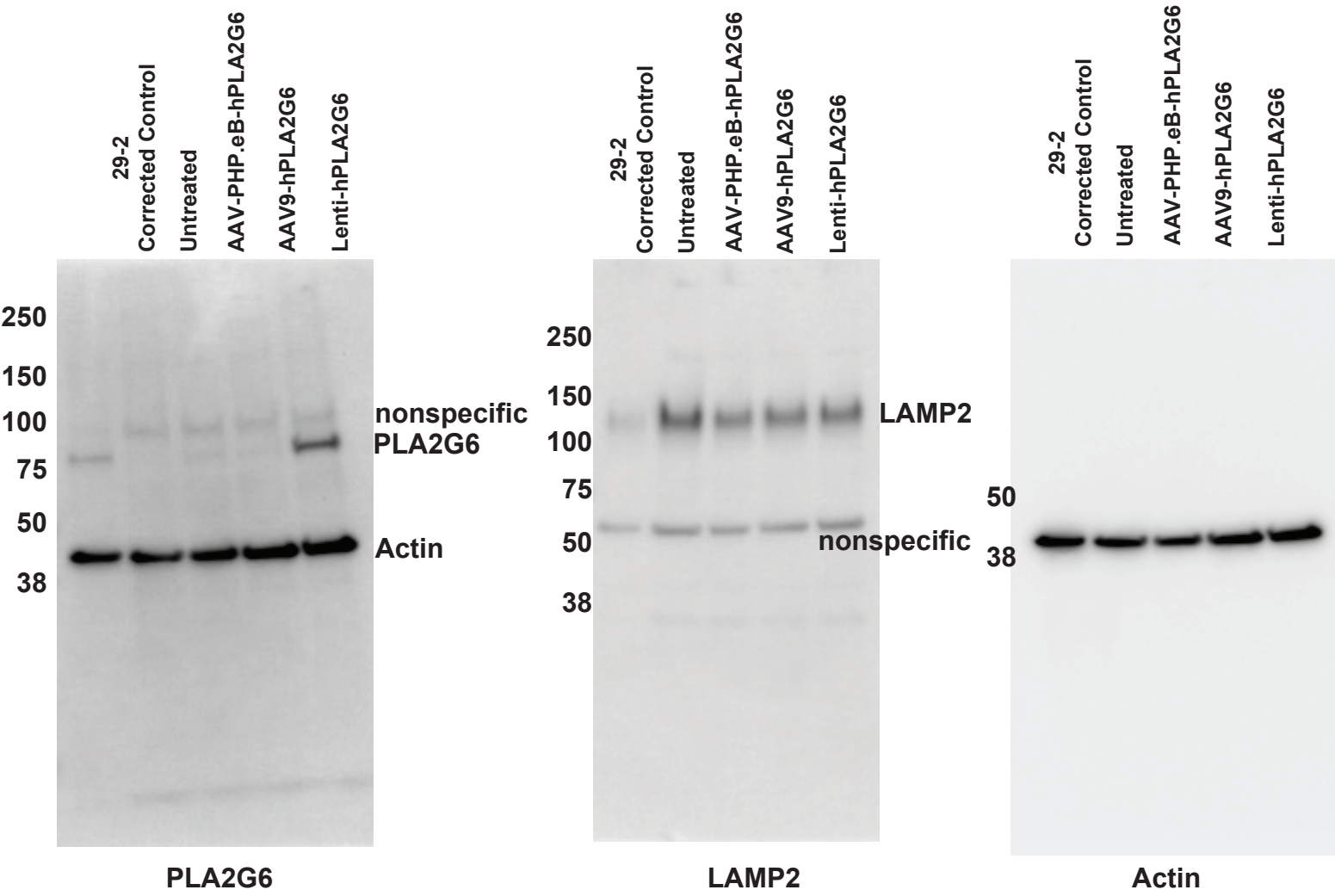

Supplement: Figure 4—source data 2. [file elife-82555-fig4-data2.pdf]
