# Supplementary material for: Exploring therapeutic strategies for infantile neuronal axonal dystrophy (INAD/PARK14)
Source: eLife. 2023 Jan 16;12:e82555. doi: 10.7554/eLife.82555 (PMC9889087; doi:10.7554/eLife.82555)

Figure 1- Figure Suppl 1- Source data

Suppl Figure 1A and 1B are the original full gel images.

Figure 1- Figure Suppl 1C

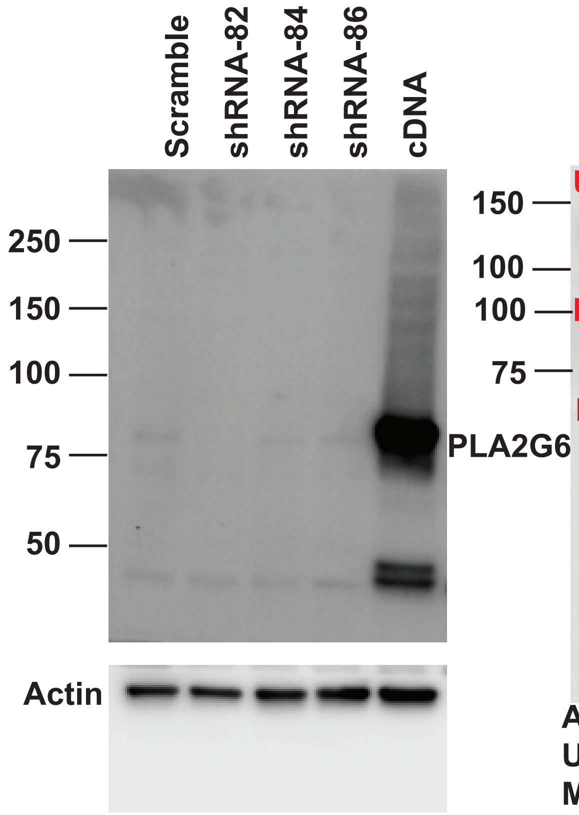

This gel were cutted in half
Upper half: PLA2G6
Bottom half: Actin

Figure 1- Figure Suppl 1E

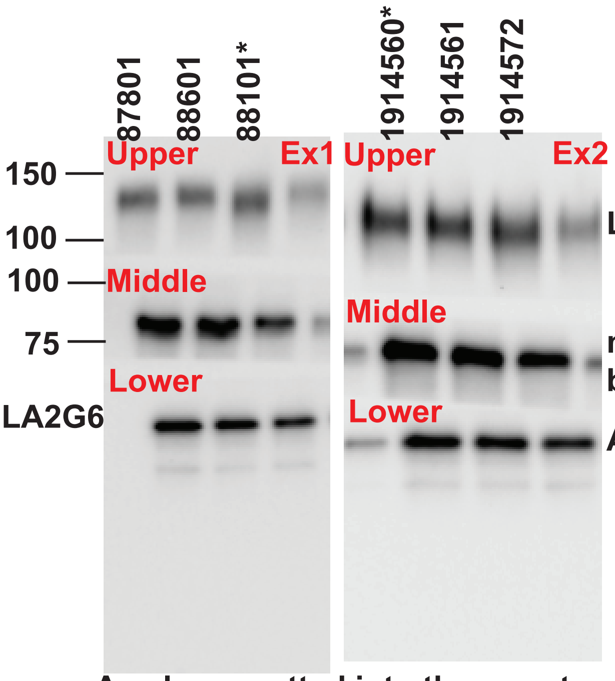

Extralanes:
Ex1: 87801 loaded in 1/3 of the amount
Ex2: 1914560 loaded in 1/3 of the amount

A gel was cutted into three parts:
Upper: LAMP2
Middle: nonspecific band detected by SAB200129
Lower: Actin
The PLA2G6 blot was redone using sc-376563 (Image below)

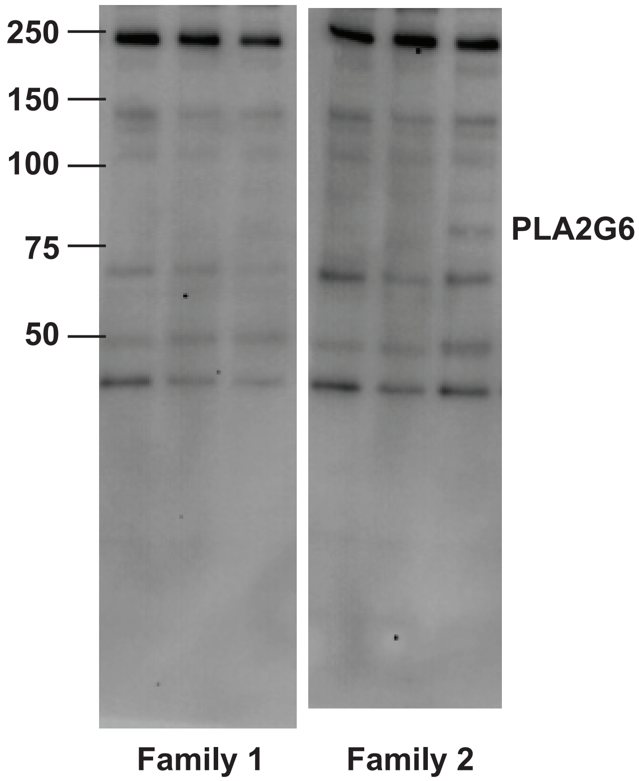

Supplement: Figure 1—figure supplement 1—source data 3. [file elife-82555-fig1-figsupp1-data3.pdf]
